# Culinary competency as a protective factor against ultra-processed food consumption and unhealthy cooking practices: a comparative study between type 1 diabetes patients and healthy controls

**DOI:** 10.3389/fnut.2026.1845185

**Published:** 2026-06-11

**Authors:** Adrián Idoate-Bayón, Aáron Quesada Hernández, Barbara Gómez-Taylor, Jorge Casaña Mohedo, Miryam Vacas-Cortés, Marina Gómez Pérez, Francisca Esteve Claramunt, Elena Sandri

**Affiliations:** 1 Diabetes Cure Research Association (DCRA), Villava, Navarra, Spain; 2 Department of Nutrition and Dietetics, Catholic University of Valencia San Vicente Mártir, Valencia, Spain; 3 Faculty of Medicine and Health Sciences, Catholic University of Valencia San Vicente Mártir, Valencia, Spain; 4 SONEV Research Group, Faculty of Medicine and Health Sciences, Catholic University of Valencia San Vicente Mártir, Valencia, Spain; 5 Department Nursing, Catholic University of Valencia San Vicente Mártir, Valencia, Spain; 6 Department of Nursing, Faculty of Nursing and Chiropody, Universitat de València, Valencia, Spain

**Keywords:** convenience foods, cooking habits, culinary competencies, fast foods, food skills, nutritional competency, self-management, type 1 diabetes

## Abstract

**Background:**

The rise of ultra-processed foods (UPFs) poses a significant challenge for chronic disease management. Culinary competence (CC) has been described as a behavioral characteristic associated with higher quality dietary patterns. This study aimed to analyze the association between CC and unhealthy dietary behaviors in individuals with type 1 diabetes (T1D) compared to a healthy population.

**Methods:**

A cross-sectional study was conducted with 592 Spanish adults (*n* = 287 T1D; *n* = 305 controls). CC was assessed using a validated 18-item scale. Statistical analyses included Spearman correlations, multiple linear regression adjusted for sociodemographic factors, and Latent Profile Analysis (LPA).

**Results:**

T1D patients reported higher CC scores than controls, particularly in health-oriented skills like label reading and recipe modification (*p* < 0.05). Multivariate models showed that CC was independently associated with lower convenience/pre-prepared food consumption in the T1D group (*B* = −0.007, *p* = 0.002). In contrast, for healthy controls, CC it was mainly associated with a reduction in unhealthy sauces and heavy cooking methods. LPA identified two distinct groups: “Culinary Experts” (72.3%) and “Moderate Competency” (27.7%). Experts showed significantly lower UPF intake regardless of clinical status or income.

**Conclusion:**

Culinary competency acts as a transversal protective factor against unhealthy eating habits. In T1D, these skills they seem to be primarily related to the avoidance of unhealthy foods. Integrating practical culinary education into clinical care could enhance self-management and metabolic health.

## 1 Introduction

Over recent decades, dietary patterns have shifted markedly across many regions of the world. Traditional food models are typically based on fresh or minimally processed ingredients and home preparation. Over time, these models have progressively given way to food environments in which convenience, speed, availability, and industrial processing play a much more prominent role. In this context, ultra-processed foods (UPFs), as defined under the NOVA classification, have become an increasingly important component of the daily diet. These products are generally described as industrial formulations made predominantly from refined substances, modified ingredients, and cosmetic additives, with little or no intact whole food remaining (1–3). This shift has been promoted by large-scale structural changes, including globalization, urbanization, industrialization of food systems, aggressive commercial expansion, and the normalization of convenience-oriented eating practices (4, 5).

The scale of this transformation is considerable. International evidence suggests that UPFs now contribute between approximately one quarter and more than one half of total daily energy intake in many populations. Particularly high values have been reported in countries such as the United States and the United Kingdom. Upward trends are also being observed in regions traditionally associated with healthier dietary patterns (2, 6, 7). Importantly, this nutritional transition does not only reflect a change in the type of products consumed. It also reflects a broader modification in the social organization of eating, including reduced time allocated to cooking, increased reliance on ready-to-eat or ready-to-heat items, and a weakening of domestic culinary practices. As a result, understanding present-day eating behaviors requires attention not only to nutrient intake, but also to the wider food environment in which food choices are made (4, 8, 9).

The growing relevance of UPFs in contemporary diets is not only a descriptive dietary trend, but also a major public health concern. Recent umbrella reviews, systematic reviews, and meta-analyses have consistently reported associations between higher convenience/pre-prepared food consumption and a broad range of adverse health outcomes (2, 3, 10–13). These outcomes include obesity, type 2 diabetes, cardiovascular disease, metabolic syndrome, certain cancers, common mental disorders, and all-cause mortality. Although the strength of associations varies depending on the outcome and study design, the overall direction of the evidence has been notably consistent across reviews. This consistency has strengthened the argument that the current food environment may be contributing to the burden of chronic disease through the growing displacement of less processed foods by industrially formulated products.

From a nutritional perspective, diets high in UPFs tend to be characterized by poorer dietary quality. These products are commonly more energy-dense and contain higher amounts of free sugars, sodium, and saturated or trans fats, while often providing lower levels of dietary fiber, protein, and micronutrients than diets based on minimally processed foods (14, 15). Proposed mechanisms linking UPFs to poorer health include excessive palatability, low satiety, altered eating speed, poorer nutrient profiles, effects on the gut microbiota, and inflammatory or metabolic disturbances associated with repeated exposure to industrial formulations (16, 17). At the same time, important methodological discussions remain regarding the heterogeneity of the NOVA classification and the extent to which causality can be inferred from predominantly observational evidence (18–20). Even so, the current evidence base is sufficiently robust to justify concern and to support the search for behavioral and environmental factors that may protect against excessive convenience/pre-prepared food consumption (2, 9, 21).

Within this context, culinary competency has emerged as a potentially relevant factor in understanding why some individuals are better able than others to maintain healthier eating habits in contemporary food environments. Culinary competency can be conceptualized as a multidimensional construct. It includes not only technical cooking ability, but also the knowledge, confidence, planning capacity, and practical decision-making required to prepare meals in daily life (22, 23). In this broader sense, it is related to constructs such as food skills, food literacy, and meal preparation autonomy. In the present study, however, the concept is specifically focused on the practical and confidence-based dimensions involved in everyday food preparation. This perspective is important because healthier eating is not determined solely by nutritional knowledge. It also depends on the practical capacity to transform intentions into everyday food choices.

Emerging evidence suggests that higher culinary competency is associated with healthier dietary behaviors. Integrative and observational studies indicate that stronger cooking skills, greater confidence in food preparation, and higher food literacy are linked to lower consumption of UPFs, greater home cooking frequency, and better adherence to healthier dietary patterns (23–28). These patterns include higher fruit and vegetable intake and stronger alignment with Mediterranean-style eating. Conversely, lower self-perceived cooking skills have been associated with greater dependence on convenience foods and more frequent consumption of ultra-processed products (25, 26). Taken together, these findings support the idea that culinary competency may function as a behavioral resource that helps individuals navigate unhealthy food environments more effectively.

The relevance of culinary competency lies not only in whether people cook, but also in how they cook and how they make food-related decisions. Preparing meals at home is often associated with better diet quality, but home cooking itself is not inherently healthy. The nutritional value of home-prepared meals depends on several factors. These include the ingredients selected, the extent to which processed products are incorporated into recipes, the use of sauces and added fats, and the methods employed during cooking. Therefore, culinary competency should not be reduced to cooking frequency alone; it may also influence the quality of food preparation practices and the level of reliance on convenience-oriented or nutritionally poorer meal solutions (22, 23, 27).

This distinction is especially relevant in current food environments, where cooking from scratch often competes with fast, highly convenient alternatives. Studies suggest that greater time spent cooking and more frequent preparation of meals at home are inversely associated with convenience/pre-prepared food consumption (26, 28). Stronger food preparation skills are linked to lower reliance on industrially formulated foods. In addition, family and community-based culinary interventions have shown promising effects on self-efficacy, healthier meal preparation, and food-related autonomy, suggesting that culinary practices may be modifiable through education and practical training (29–31). Accordingly, culinary competency may represent more than a domestic skill; it may operate as a protective behavioral factor influencing both food choice and the practical implementation of healthier eating.

The possible protective role of culinary competency may be particularly relevant in individuals living with type 1 diabetes (T1D). In this population, food-related decisions are relevant not only for general health promotion but also form part of daily self-management. Meal planning, food selection, interpretation of labels, and practical food preparation can all influence glycaemic management, treatment adherence, and confidence in handling everyday eating situations. For that reason, the ability to prepare meals and make informed food choices may be especially valuable in T1D, where nutrition is closely linked to self-care and autonomy (32, 33).

Although the literature is still limited compared with that available in the general population or in type 2 diabetes, some emerging evidence suggests that culinary and food-related educational approaches may be beneficial in people with diabetes. Pilot and educational studies have reported improvements in self-efficacy, healthy meal preparation, and food-related psychosocial outcomes after culinary medicine or cooking-based interventions (31, 33, 34). In addition, observational work has explored cooking habits and food choices among individuals with T1D, pointing to the relevance of practical food behaviors in this group (32). These findings suggest that culinary competency may have a particularly meaningful role in T1D. It may contribute to healthier dietary patterns and may also support greater independence and confidence in daily disease management.

### 1.1 Study hypotheses

Based on the existing literature regarding the role of food literacy in chronic disease management and the nutritional transition toward convenience-oriented diets, this study tests the following hypotheses:

*H1:* Individuals with type 1 diabetes (T1D) will exhibit significantly higher culinary competency scores than healthy controls, particularly in health-oriented domains, due to the continuous demands of glycemic self-management.

*H2:* Higher culinary competency will act as a robust independent protective factor against the consumption of convenience/pre-prepared food across the total population, regardless of socioeconomic status.

*H3:* The practical application of culinary skills will differ by clinical status: in the T1D group, competency will primarily predict the avoidance of convenience/pre-prepared food, while in healthy controls, it will primarily predict the technical refinement of cooking methods and reduction of unhealthy additives.

*H4:* Latent Profile Analysis will identify a distinct ‘Culinary Expert’ subgroup that demonstrates significantly better dietary quality indicators compared to those with moderate competency.

## 2 Materials and methods

### 2.1 Study design

This research was designed as a cross-sectional, observational, and descriptive study.

### 2.2 Population and recruitment

The target population comprised adults living in Spain. To determine the necessary sample size, statistical data were drawn from the National Institute of Statistics (INE) for the general population and the Spanish Diabetes Society (SED) (35), which reports an estimated 90,000 people living with type 1 diabetes (T1D) in the country. Utilizing a 95% confidence interval, a 7% margin of error, and a maximum variability assumption (*p* = *q* = 0.5), the initial target was set at a minimum of 196 individuals. Through a combination of convenience and “snowball” sampling techniques (36), a final cohort of *N* = 592 was recruited, consisting of *n* = 305 healthy controls and *n* = 287 patients with T1D.

### 2.3 Eligibility criteria

Participants were required to meet the following Inclusion Criteria:

- Attainment of adulthood (18 years of age or older).
- Spanish nationality and current residence within Spain.
- For the experimental group, a confirmed T1D diagnosis of at least 1 year’s duration.

Conversely, the following Exclusion Criteria were applied:

- Circumstances involving restricted dietary control, such as incarceration or current hospitalization.
- Cognitive or physical impairments that interfere with the ability to cook.

The presence of medical conditions (excluding T1D in the clinical group) that necessitate a strictly regulated diet.

### 2.4 Ethical considerations and data collection

Data were collected between June 2025 and January 2026. Recruitment was facilitated via the researchers’ social networking profiles and the formal communication platforms of the Diabetes Cure Research Association (DCRA).

The study protocol received formal approval from Ethics Research Committee of the Catholic University of Valencia (approval number UCV/2024-2025/144, June 4, 2025). Prior to beginning the survey, all subjects provided informed consent. The study was conducted in full accordance with EU Regulation 2016/679 (GDPR), guaranteeing participant anonymity and the confidential handling of data.

### 2.5 Assessment tool

Culinary proficiency was evaluated using a structured questionnaire, which was previously translated and culturally adapted for the Spanish context by researchers at Blanquerna University (37). This instrument is based on an original English-language tool validated in Ontario (38). Following the research framework established by Sandri for the Spanish population, the survey was expanded with seven qualitative items (39). These additional questions, developed in consultation with subject-matter experts, were designed to provide a deeper characterization of dietary patterns and daily cooking routines. Furthermore, a comprehensive sociodemographic profile was obtained for all study participants.

In the present study, the scale demonstrated excellent internal consistency across the entire sample (*N* = 592 Spanish adults) (alpha = 0.94). To ensure the instrument’s stability for comparative analysis, reliability was also calculated separately for both study cohorts, yielding high internal consistency in both the type 1 diabetes group (*n* = 287) (alpha = 0.94) and the healthy control group (*n* = 305) (alpha = 0.93). Following the previous validation of the scale in the Spanish population, the instrument is organized unidimensionally to obtain a global score. However, it conceptually groups skills into four key dimensions:

- Technical Skills: Items 1–3, 5–6, 15–16, and 18.
- Health and Nutrition Skills: Items 13 and 17.
- Planning and Management: Items 4, 12, and 14.
- Preparation of Specific Foods: Items 7–11.

### 2.6 Variables and measurements

#### 2.6.1 Sociodemographic indicators

The study analyzed several sociodemographic factors:

- Sex: Categorized dichotomously (male/female).
- Age: Stratified into young adults (18–30 years) and adults (>30 years).
- Education: Grouped into basic education (encompassing primary/secondary schooling, high school, or vocational training) and higher education (comprising university, Master’s, or Doctoral degrees).
- Income: Classified by monthly household net earnings into low income (≤2,200 euros), medium–high income (>2,200 euros), or “not reported.”
- Employment Status: Recorded as employed, unemployed, student, retired, or otherwise not in the workforce.

#### 2.6.2 Clinical and diabetes-specific data

A specialized section of the instrument targeted the clinical history of participants with diabetes, assessing the following:

- Disease Duration: Calculated as the total number of years since the initial diagnosis.
- Treatment Regimen: Participants identified their current therapy from a list including oral antidiabetic agents (e.g., metformin, SGLT2 inhibitors, GLP-1 agonists, or DPP-4 inhibitors), insulin therapy (via multiple daily injections or continuous subcutaneous infusion/pumps), or a combination of both.
- Monitoring Technology: Identified the primary method for glucose tracking, distinguishing between traditional capillary meters, standalone continuous/flash glucose monitors (CGM/FGM), and sensors integrated with insulin pump systems.
- Metabolic Management: Glycated Hemoglobin (HbA1c) levels were used to gauge glycemic control. Following clinical standards (40), results were dichotomized into “adequate control” (HbA1c < 7.0%) or “suboptimal control” (HbA1c ≥ 7.0%). Additionally, Time in Range (TIR) was recorded, with a value > 70% serving as the clinical threshold for optimal management.

#### 2.6.3 Culinary habits

Participants’ cooking practices were evaluated through seven specific items. While the sources for recipe consultation were gathered qualitatively, the remaining variables were quantitatively categorized to reflect dietary quality. Home cooking frequency was measured on a 4-point scale ranging from 0 (“I do not normally cook”) to 3 (“Every day or almost every day”). Convenience food consumption was recorded based on weekly intake, scored from 0 (“I do not know”) and 1 (“None”) to 4 (“More than 5 times”). It is important to clarify that this measure was assessed as a behavioral indicator of reliance on industrially prepared and ready-to-eat meals, rather than a strict nutritional classification based on the NOVA framework. In this context, ‘convenience/pre-prepared food’ serve as a proxy for the frequency of use of pre-prepared food products in the domestic environment. The remaining variables, including the use of healthy appliances, healthy cooking techniques, unhealthy techniques, and the addition of unhealthy condiments or sauces, were measured using a 5-point Likert scale (1 = Never to 5 = Always). For analytical purposes, these behaviors were categorized to reflect dietary quality and health-promoting versus adverse health practices.

#### 2.6.4 Assessment of culinary competency

Culinary proficiency was measured through a series of tasks where participants self-reported their confidence levels on a five-point Likert scale (ranging from 1, “very low confidence,” to 5, “very high confidence”). The summation of these items resulted in a global culinary competency score, with potential values spanning from a minimum of 18 to a maximum of 90 points.

### 2.7 Statistical analysis

Following the data collection phase, all survey responses were organized in a Microsoft Excel database. A rigorous screening process was conducted to identify and rectify anomalies, focusing on data entry errors and extreme outliers. Specific variables were recoded or derived from the raw data to facilitate analysis. To ensure the reliability of the anthropometric results, Body Mass Index (BMI) records deemed clinically implausible (those < 14 or > 40 kg/m^2^) were excluded from the final dataset.

The refined database was then transferred to Jamovi (version 2.3.28.0) for formal processing (41). The normality of the data distribution was tested using the Shapiro–Wilk method, which revealed that the variables did not follow a normal distribution. These findings were further validated through the visual inspection of Q–Q plots (42).

Consequently, non-parametric statistical methods were employed for the analysis. The Chi-square test was used to identify associations between categorical variables. For comparisons between the T1D group and the healthy control group regarding continuous or ordinal variables, the Mann–Whitney U test was applied. Furthermore, multiple linear regression models were developed to investigate the independent predictors of cooking behaviors and culinary skills, adjusting for socioeconomic and demographic covariates. The correlation between the frequency of convenience food intake and culinary competence was evaluated using Spearman’s rank correlation coefficient (ƍ).

Statistical significance was defined by a *p*-value threshold of 0.05. Descriptive data are presented as absolute and relative frequencies for categorical variables, while continuous data are summarized using both means (with standard deviations) and medians (with interquartile ranges).

A Latent Profile Analysis (LPA) was conducted to identify unobserved subgroups (latent profiles) of participants based on their culinary competencies (43, 44). Unlike traditional clustering methods, LPA is a model-based approach that provides fit indices to determine the optimal number of classes. This method has been increasingly utilized in nutritional and health research to identify complex behavioral patterns (45). The best-fitting model was selected using the Bayesian Information Criterion (BIC) (46), where lower values indicate a more parsimonious fit, and Entropy, which measures the precision of participant assignment (values > 0.80 indicate good separation). Additionally, the Bootstrapped Likelihood Ratio Test (BLRT) was used to compare the k-class model against a k-1 model. According to these criteria, a two-class solution (Model 3) was identified as the most robust, allowing for different variances across profiles. Missing values in the culinary skill indicators were handled using median imputation to maintain the integrity of the sample (*N* = 592). Statistical analyses were performed using Jamovi (version 2.3) with the snowRMM and tidyLPA modules.

The selection of covariates for the multivariate regression models (age, sex, income, and education) was based on their documented influence on dietary habits and culinary skills in previous literature (23–28, 39). While interaction effects between group (T1D vs. healthy) and culinary competency were initially considered, the study focused on the independent predictive value of culinary skills within each cohort to identify distinct behavioral patterns. Consequently, separate models were prioritized to facilitate the clinical interpretation of results for each population.

## 3 Results

### 3.1 Profile of the study population

The clinical and demographic characteristics of the total sample (*N* = 592) are detailed in Table 1, distinguishing between the type 1 diabetes cohort (*n* = 287) and the healthy control group (*n* = 305). Female participants represented the majority in both subsets, accounting for 76.7% of the T1D group and 80.3% of the healthy participants. Age distribution varied significantly between the two groups. Those with T1D were generally older, with a mean age of 40.1 ± 14.0 years, compared to 34.4 ± 15.5 years in the control group. This disparity is further evidenced by the age stratification: 68.3% of the T1D group were over the age of 30, whereas only 47.2% of the healthy controls fell into this category. Consequently, the healthy group featured a markedly higher percentage of students (45.6%) than the clinical group (11.8%). Regarding socioeconomic status, nearly half of the T1D participants (48.1%) reported being in the low-income bracket. In contrast, the healthy group was characterized by a higher economic standing, with 50.8% reporting medium-to-high income levels. The clinical profile of the T1D cohort revealed an average disease duration of 19.2 ± 12.9 years, with diagnosis dates ranging from 1970 to 2025. Metabolic management, measured through Glycated Hemoglobin (HbA1c) levels from the preceding trimester, yielded a mean of 7.29 ± 1.28. Individual HbA1c values within this group showed significant variance, ranging from a minimum of 4% to a maximum of 13%.

**Table 1:** Demographic and clinical characteristics of the study population (*N* = 592; T1D, *n* = 287; Healthy group, *n* = 305).

Variables	Mean ± SD or *N* (%)	
	TD1 patients (*n* = 287)	Health population (*n* = 305)
Sex		
Male	67 (23.3%)	60 (19.7%)
Female	220 (76.7%)	220 (80.3%)
Age (years)	40.1 ± 14.0	34.4 ± 15.5
Median (Interquartile range)	37 ± 18.9	28 ± 17.3
Min	18	18
Max	74	87
Young (18–30 years)	91 (31.7%)	161 (52.8%)
Adults (>30 years)	196 (68.3%)	144 (47.2%)
Education level		
Basic education	127 (44.3%)	160 (52.6%)
Higher education	160 (55.7%)	145 (47.4%)
Income level		
Low	138 (48.1%)	80 (26.2%)
Medium-high	111 (38.7%)	155 (50.8%)
Do not know-no answer	38 (13.2%)	70 (23.0%)
Work situation		
Employed	201 (70.0%)	140 (45.9%)
Unemployed	23 (8.0%)	14 (4.6%)
Student	34 (11.8%)	139 (45.6%)
Retired	20 (7.0%)	4 (14.4%)
Do not work	9 (3.1%)	8 (2.6%)
Year of diabetes diagnosis	2007 ± 12.9	
Median (Interquartile Range)	2008 ± 17.4	
Min	1970	
Max	2025	
Years since diabetes diagnosis	19.2 ± 12.9	
Median (Interquartile Range)	18 ± 17.4	
Min	1	
Max	56	
HbA1c value during the last 3 months (%)	6.8 ± 1.2	
Median (Interquartile Range)	6.7 ± 1.6	
Min	4.2	
Max	13.0	
Time in range TIR (%)	78.6 ± 12.3	
Median (Interquartile Range)	80.0 ± 16.3	
Min	30	
Max	100	

Regarding the management of type 1 diabetes (T1D), insulin-centered regimens were the primary therapeutic approach. Most of the cohort (54.0%) utilized multiple daily injections for insulin delivery, while a significant segment (41.8%) employed continuous subcutaneous insulin infusion via pump devices. Supplemental therapy involving oral glucose-lowering medications—including metformin, SGLT2 inhibitors, or GLP-1 receptor agonists—remained rare. These adjunct treatments were used in conjunction with insulin injections by only 3.1% of participants and alongside insulin pump therapy by a mere 1.0%.

In terms of metabolic monitoring, the study population showed a high level of integration with digital health technologies. The most common tool for tracking glucose levels was the use of standalone flash or continuous glucose monitoring (CGM/FGM) systems, utilized by 57.8% of the sample. Additionally, 40.1% of participants used glucose sensors that were directly integrated with their insulin pump systems. Traditional capillary blood glucose monitoring was notably infrequent, serving as the primary tracking method for only 2.1% of the respondents.

### 3.2 Comparison of culinary competencies between T1D patients and healthy controls

The descriptive analysis of self-reported culinary skills indicated that individuals with type 1 diabetes (T1D) showed overall higher competency levels than the healthy control group, reflected in a greater Total Culinary Education Score (69.33 ± 16.61 vs. 66.20 ± 15.40, respectively) (see Table 2).

**Table 2:** Comparison of cooking habits and culinary competencies between T1D patients and the healthy population.

Culinary competencies	TD1 patients (*n* = 287)				Health population (*n* = 305)			
	Mean	SD	Median	IQR	Mean	SD	Median	IQR
Preparatory techniques	4.20	1.17	5.00	1.00	4.15	1.07	5.00	1.44
Culinary techniques	4.22	0.99	5.00	1.00	4.10	1.07	5.00	1.44
Use of kitchen equipment	4.25	0.99	5.00	1.00	4.08	1.05	5.00	1.42
Food preservation	3.68	1.21	4.00	2.00	3.45	1.29	5.00	1.74
Recognize Firing Point	3.75	1.16	4.00	2.00	3.49	1.19	5.00	1.61
Food safety handling	4.08	1.06	4.00	2.00	3.89	1.04	5.00	1.40
Cooking cereals	4.05	1.11	4.00	2.00	4.22	1.02	5.00	1.38
Cooking vegetables	4.38	0.94	5.00	1.00	4.14	1.09	5.00	1.47
Cooking protein foods	4.33	0.93	5.00	1.00	4.16	1.05	5.00	1.42
Cooking a balanced meal	4.29	0.91	5.00	1.00	4.02	1.07	5.00	1.44
Cooking of different foods	4.32	0.98	5.00	1.00	4.07	1.11	5.00	1.50
Compare food prices	3.94	1.07	4.00	2.00	3.88	1.06	5.00	1.43
Read nutritional information	4.17	1.09	5.00	1.00	3.70	1.28	5.00	1.73
Weekly meal planning	3.40	1.31	4.00	3.00	3.24	1.34	5.00	1.81
Read recipes	3.89	1.17	4.00	2.00	3.70	1.26	5.00	1.70
Modify recipe (missing ingredient)	4.04	1.05	4.00	2.00	3.82	1.24	5.00	1.67
Modify recipe (healthier)	4.13	1.11	5.00	1.25	3.76	1.28	5.00	1.73
Using leftovers	3.97	1.18	4.00	2.00	3.77	1.24	5.00	1.67
Total culinary education	69.33	16.61	72.00	22.00	66.20	15.40	90.00	20.78

In particular, the T1D group demonstrated stronger performance in health-related competencies, including reading nutritional information (4.17 ± 1.09 vs. 3.70 ± 1.28), modifying recipes to improve their nutritional quality (4.13 ± 1.11 vs. 3.76 ± 1.28), and preparing vegetables (4.38 ± 0.94 vs. 4.14 ± 1.09). Both groups reported high confidence in basic preparatory and general culinary techniques. The only domain in which the healthy group scored slightly higher was cooking cereals (4.22 ± 1.02 vs. 4.05 ± 1.11). Weekly meal planning emerged as the lowest-rated competency in both groups (3.40 ± 1.31 in T1D and 3.24 ± 1.34 in controls).

Overall, these results suggest that the routine demands of diabetes self-management may contribute to the development of more advanced culinary and nutrition-related skills, particularly those linked to health-oriented food choices and dietary literacy.

### 3.3 Relationship between culinary competency and unhealthy dietary habits

Statistical analysis using Spearman’s rank correlation revealed that higher levels of culinary competency were significantly associated with better dietary quality indicators across the total population (Table 3). Specifically, increased cooking skills correlated with a lower frequency of convenience/pre-prepared consumption (ƍ = −0.094, *p* = 0.022), reduced use of unhealthy sauces and fats (ƍ = −0.126, *p* = 0.002), and a lower reliance on heavy cooking methods such as frying (ƍ = −0.127, *p* = 0.002). Subgroup analysis showed distinct behavioral patterns. In the T1D cohort, culinary competency was a significant predictor for avoiding convenience/pre-prepared foods (ƍ = −0.142, *p* = 0.016), yet it showed no significant correlation with the use of sauces or heavy cooking techniques. This suggests that dietary restrictions inherent to diabetes management may already minimize these practices. Conversely, in the healthy control group, culinary skills were linked to a reduced intake of unhealthy sauces (ƍ = −0.171, *p* = 0.003) and heavy cooking methods (ƍ = −0.131, *p* = 0.022). However, skills did not significantly influence the consumption of convenience/pre-prepared foods in this group (*p* = 0.548), indicating that healthy individuals may prioritize cooking techniques over the total avoidance of pre-prepared products.

**Table 3:** Spearman’s rank correlations between total culinary competency and dietary quality indicators by study group.

Population group	Variable	Correlation (*ρ*)	*p*-value	Significance
Total population (*N* = 592)	Convenience/pre-prepared food	**−0.094**	0.022	Significant
	Unhealthy sauces/fats	**−0.126**	0.002	Highly significant
	Heavy cooking methods	**−0.127**	0.002	Highly significant
Type 1 diabetes (*n* = 287)	Convenience/pre-prepared food	**−0.142**	0.016	Significant
	Unhealthy sauces/fats	−0.040	0.501	Not significant
	Heavy cooking methods	−0.078	0.187	Not significant
Healthy controls (*n* = 305)	Convenience/pre-prepared food	−0.034	0.548	Not significant
	Unhealthy sauces/fats	**−0.171**	0.003	Highly significant
	Heavy cooking methods	**−0.131**	0.022	Significant

The values in bold are statistically significant.

**Figure 1:**
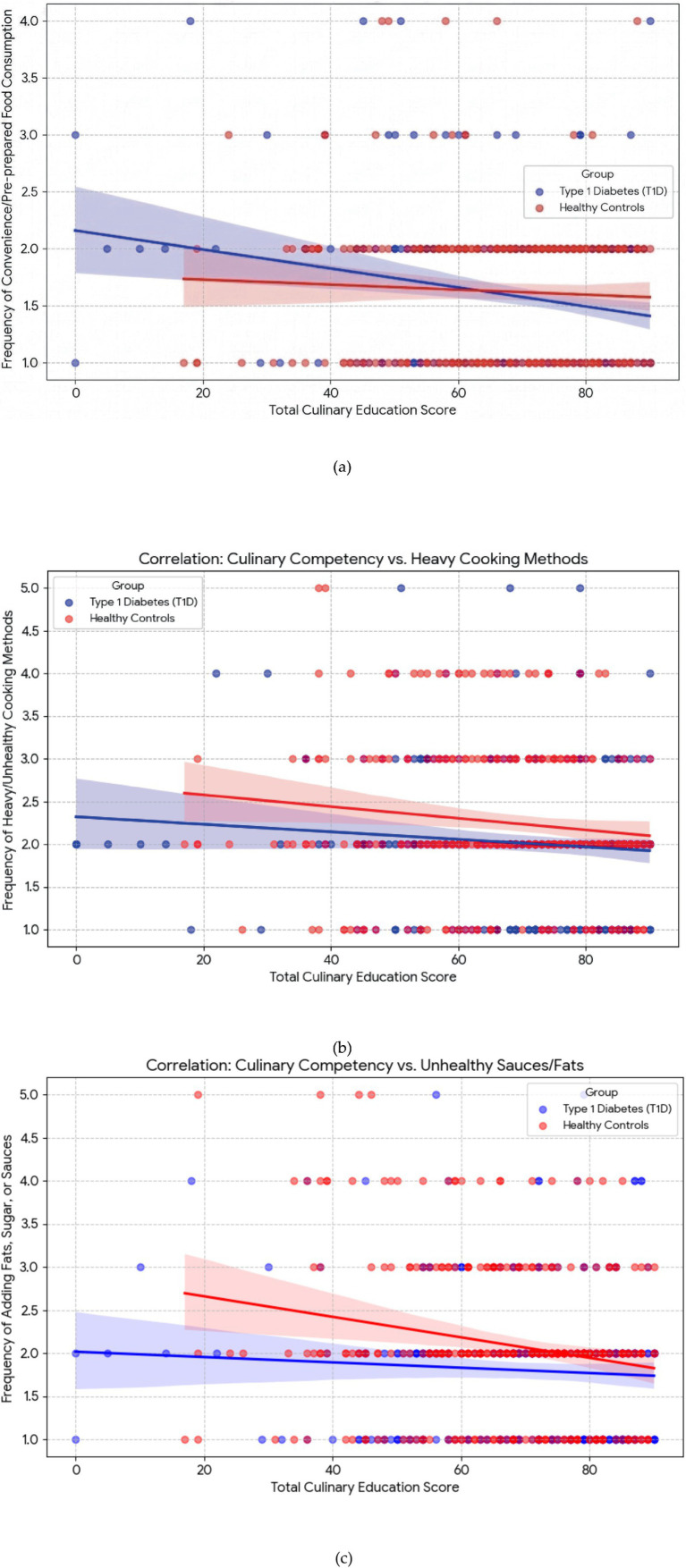
**(a)** Correlation between culinary competency and convenience food consumption. (**b**) Correlation between culinary competency and unhealthy sauces/fats. **(c)** Correlation between culinary competency and heavy cooking methods. Blue: Type 1 diabetes cohort, Red: healthy controls.

The visual analysis of the correlation scatterplots (Figures 1a–c) further elucidates the divergent impact of culinary competency on dietary behaviors across the two study groups. Figure 1a illustrates that for individuals with type 1 diabetes (blue line), higher culinary proficiency is specifically associated with a more pronounced reduction in the frequency of convenience/pre-prepared food consumption compared to the healthy cohort (red line), indicating that this clinical group leverages cooking skills as a primary strategic tool to avoid convenience/pre-prepared products.

In contrast, Figures 1b,c reveal that in the healthy control group (red line), culinary competency serves primarily to refine dietary quality by significantly decreasing the use of unhealthy sauces, fats, and heavy cooking techniques (e.g., frying). For the T1D group (blue line), the regression slopes for sauces and heavy methods are notably flatter, suggesting that their consumption of these items remains consistently low regardless of their perceived skill level, likely due to the foundational dietary education received as part of standard diabetes management. These visual trends confirm that while culinary competency is protective for both groups, its practical application shifts from ‘avoidance of processed foods’ in T1D patients to ‘technical refinement and fat reduction’ in the healthy population.

### 3.4 Impact of culinary competency adjusted for sociodemographic factors

To isolate the specific contribution of culinary competency to dietary quality, a series of multiple linear regression models were conducted (Table 4). These models adjusted for age, sex, income, and educational level. Even after controlling these variables, culinary competency remained a statistically significant predictor of cooking behaviors and dietary choices.

**Table 4:** Multivariate linear regression analysis of culinary competency as a predictor of dietary habits and cooking methods, adjusted for sociodemographic factors and educational attainment.

Dependent variable	Population group	*B* (SE)	95% CI	*p*-value	*R* ^2^
Convenience/pre-prepared food	Total population	−0.005 (0.002)	**[−0.008, −0.002]**	0.003**	0.068
	Type 1 diabetes (T1D)	−0.007 (0.002)	**[−0.011, −0.003]**	0.002**	0.060
	Healthy controls	−0.002 (0.002)	[−0.006, 0.002]	0.340	0.101
Unhealthy sauces/fats	Total population	−0.008 (0.002)	**[−0.012, −0.004]**	<0.001***	0.104
	Type 1 diabetes (T1D)	−0.003 (0.003)	[−0.009, 0.003]	0.354	0.065
	Healthy controls	−0.014 (0.003)	**[−0.020, −0.008]**	<0.001***	0.129
Heavy cooking methods	Total population	−0.006 (0.002)	**[−0.010, −0.002]**	0.004**	0.078
	Type 1 diabetes (T1D)	−0.004 (0.003)	[−0.010, 0.002]	0.189	0.073
	Healthy controls	−0.009 (0.003)	**[−0.016, −0.002]**	0.008**	0.059

*B* = Unstandardized coefficient; *R*^2^ = Coefficient of determination. All models were adjusted for age, sex, net monthly income, and educational attainment. UPF, Ultra-processed foods. Significant results: **p < 0.01, ***p < 0.001. The values in bold are statistically significant.

For the type 1 diabetes (T1D) cohort, the model demonstrated that culinary skills are a robust independent predictor for reducing the consumption of Convenience/pre-prepared food (*B* = −0.007, *p* = 0.002). This suggests that within the clinical population, higher cooking proficiency is a key tool for avoiding ultra-processed products, regardless of the patient’s formal education level or socioeconomic background. In the Healthy Control group, the influence of culinary competency was predominantly seen in the reduction of Unhealthy Sauces and Fats (*B* = −0.014, *p* < 0.001) and Heavy Cooking Methods (*B* = −0.009, *p* = 0.008). While higher educational levels contributed to the overall explanatory power of the models (*R*^2^ values increased across all healthy group models), culinary competency maintained its role as a primary independent driver of healthier cooking practices. These results highlight that teaching practical kitchen skills is a valuable intervention for the general public that goes beyond general academic or nutritional knowledge. As shown in Table 4, the 95% CI for the T1D group [−0.011, −0.003] confirms that higher CC is consistently associated with lower convenience/pre-prepared food consumption intake.

### 3.5 Latent Profile Analysis (LPA) of culinary competency

To identify distinct subgroups within the study population based on culinary competency, a Latent Profile Analysis (LPA) was conducted using 18 specific culinary and food-related indicators. Following a systematic comparison of competitive models, Model 3 with a two-class solution was identified as the optimal fit for the data. This selection was supported by the lowest Bayesian Information Criterion (BIC = 19,233) and confirmed by an Analytic Hierarchy Process (AHP) based on AIC, BIC, and SABIC indices.

The classification quality was highly robust, as indicated by an Entropy value of 0.910 (for Model 3) and 0.966 (for the final refined estimation), suggesting excellent separation and minimal overlap between the identified latent classes. The Bootstrapped Likelihood Ratio Test (BLRT) further supported the superiority of the two-class model over a single-class solution (*p* = 0.0099). Although a three-cluster solution was initially explored, the Latent Profile Analysis (LPA) indicated that a two-class model (Model 3) provided the most parsimonious and statistically superior fit.

**Figure 2:**
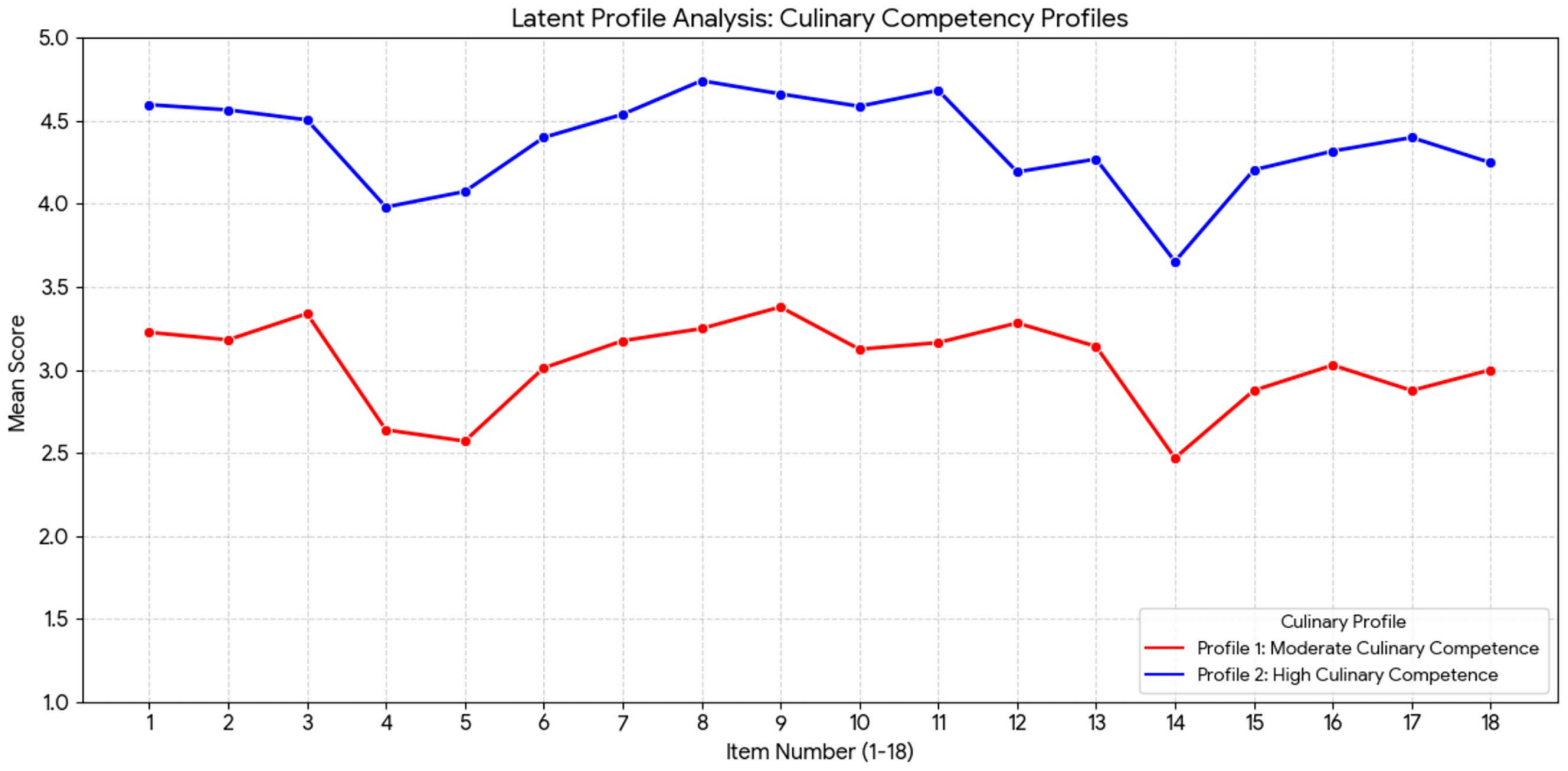
Mean scores for culinary competency items by latent profile. The *x*-axis represents the 18 culinary skill items: (1) Preparatory techniques, (2) culinary techniques, (3) use of kitchen equipment, (4) food preservation, (5) recognize firing point, (6) food safety handling, (7) cooking cereals, (8) cooking vegetables, (9) cooking protein foods, (10) cooking a balanced meal, (11) cooking of different foods, (12) compare food prices, (13) read nutritional information, (14) weekly meal planning, (15) read recipes, (16) modify the recipe if an ingredient is missing, (17) modify recipe to make it healthier, (18) using leftovers.

The two latent profiles represent distinct tiers of culinary proficiency and strategic food management (see Table 5 and Figure 2):

- Profile 1: Moderate Culinary Competency (*n* ≈ 27.7% of the sample): This group exhibited significantly lower mean scores across all culinary domains. The most pronounced deficits were observed in strategic organizational skills, specifically in Weekly meal planning (Mean = 2.49) and Food preservation (Mean = 2.58). This profile reflects individuals with basic technical knowledge but limited autonomy in complex food management.
- Profile 2: High Culinary Competency (*n* ≈ 72.3% of the sample): Labeled as “Culinary Experts,” this majority group demonstrated high proficiency in both technical and cognitive culinary tasks. Maximal scores were observed in Cooking vegetables (Mean = 4.73), Cooking cereals (Mean = 4.57), and Cooking protein foods (Mean = 4.65). Notably, this group also excelled in health-oriented tasks, such as Modifying recipes to make them healthier (Mean = 4.42).

**Table 5:** Culinary competence according to latent profiles.

Culinary competence item	Profile 1 Mean (SD)	Profile 2 Mean (SD)	Effect size (*d*)	*p*-value
1. Preparatory techniques	3.18 (0.72)	4.64 (0.72)	2.01	<0.001
2. Culinary techniques	3.13 (0.61)	4.59 (0.61)	2.41	<0.001
3. Use of kitchen equipment	3.28 (0.62)	4.56 (0.62)	2.06	<0.001
4. Food preservation	2.58 (1.11)	3.93 (1.11)	1.21	<0.001
5. Recognize firing point	2.51 (0.86)	4.06 (0.86)	1.81	<0.001
6. Food safety handling	2.90 (0.64)	4.40 (0.64)	2.35	<0.001
7. Cooking cereals	3.16 (0.63)	4.57 (0.63)	2.27	<0.001
8. Cooking vegetables	3.08 (0.45)	4.73 (0.45)	3.68	<0.001
9. Cooking protein foods	3.22 (0.54)	4.65 (0.54)	2.63	<0.001
10. Cooking a balanced meal	2.98 (0.45)	4.60 (0.45)	3.64	<0.001
11. Cooking of different foods	2.99 (0.52)	4.64 (0.52)	3.19	<0.001
12. Compare food prices	3.18 (0.92)	4.21 (0.92)	1.12	<0.001
13. Read nutritional information	3.10 (1.13)	4.26 (1.13)	1.03	<0.001
14. Weekly meal planning	2.50 (1.41)	3.71 (1.41)	0.86	<0.001
15. Read recipes	2.90 (1.01)	4.26 (1.01)	1.35	<0.001
16. Modify recipe (missing ingredient)	3.03 (0.91)	4.32 (0.91)	1.42	<0.001
17. Modify recipe (healthier)	2.80 (0.84)	4.42 (0.84)	1.93	<0.001
18. Using leftovers	2.98 (1.05)	4.28 (1.05)	1.24	<0.001

*d* = Cohen’s *d* effect size. Values > 0.8 represents a large effect. All comparisons were statistically significant at *p* < 0.001.

Statistical comparisons showed significant differences (*p* < 0.001) for every indicator between the two profiles. The high classification probability (Prob_max = 0.99) confirms that these profiles are distinct and reliable for further analysis regarding their association with clinical status (type 1 diabetes) and dietary outcomes, such as the consumption of convenience/pre-prepared foods.

### 3.6 Sociodemographic and behavioral characterization of latent profiles

A comparative analysis was conducted to examine the differences between the two identified culinary profiles (Tables 6, 7). The results indicate that the ‘Culinary Expert’ profile is primarily distinguished by its specific protective effect on convenience/pre-prepared food consumption rather than by broad sociodemographic disparities.

**Table 6:** Sociodemographic and clinical characteristics by culinary latent profile.

Membership	*N*	%	*N*	%	*p*-value
	Male		Female		
Profile 1	66	15.60%	51	12.10%	**<0.001**
Profile 2	61	14.40%	245	57.90%	
	Young (≤30 years)		Adults (>30 years)		
Profile 1	58	13.70%	59	13.90%	0.320
Profile 2	155	36.60%	151	35.70%	
	Basic education		DT1		
Profile 1	58	13.70%	59	13.90%	0.843
Profile 2	155	36.60%	151	35.70%	
	Healthy population		DT1		
Profile 1	61	14.40%	56	13.20%	0.568
Profile 2	169	40.00%	137	32.40%	

Data represent Mean (SD) or percentages (%). *p*-Values indicate differences between Profile 1 (Moderate) and Profile 2 (Expert). Statistically significant differences (*p* < 0.05) were observed only for Age. The values in bold are statistically significant.

**Table 7:** Dietary habit frequencies across culinary latent profiles.

Dietary habit item	Profile 1 Mean (SD)	Profile 2 Mean (SD)	95% CI (Difference)	*p*-value
Convenience/pre-prepared food	1.74 (0.62)	1.59 (0.64)	**[1.53, 1.65]**	0.022*
Unhealthy sauces/fats	2.13 (1.05)	1.94 (0.85)	[−0.34, 0.04]	0.201
Heavy cooking methods	2.26 (0.89)	2.20 (0.87)	[−0.22, 0.10]	0.445

Lower values indicate healthier, less frequent consumption. A significant difference was confirmed for Convenience/pre-prepared Food Consumption (*p* < 0.05). CI, Confidence Interval for Profile 2 means. The values in bold are statistically significant.

Statistically significant differences were observed in the consumption of convenience/pre-prepared food (*p* < 0.001), where participants in the Expert profile reported a significantly lower frequency of intake (Mean = 1.53) compared to those in the Moderate profile (Mean = 1.84). Additionally, Age was the only sociodemographic variable that reached statistical significance (*p* < 0.05), suggesting that culinary expertise may be partially associated with life stage or accumulated experience.

In contrast, no statistically significant differences were found between profiles regarding sex, education level, household income, or diabetes status (*p* > 0.05). Furthermore, while the Expert group showed descriptively lower scores for the use of unhealthy sauces and heavy cooking methods, these differences did not reach the threshold for statistical significance in this model. These findings suggest that while high culinary competency is an independent predictor of reduced convenience/pre-prepared food consumption, its development and benefits are distributed transversally across the different clinical and socioeconomic groups studied.

## 4 Discussion

This study confirms that culinary competence is a key determinant of dietary quality, with a significant impact on reducing convenience/pre-prepared food consumption, and adopting healthier cooking practices. Multivariate analyses, adjusted for age, sex, income, and educational level, showed that culinary competence remains an independent predictor of eating habits, indicating that its influence transcends sociodemographic factors. In the cohort with type 1 diabetes (T1D), these skills were robustly associated with lower convenience/pre-prepared food consumption, while in the healthy population their effect focused on reducing the use of unhealthy sauces and intensive cooking methods. This finding suggests that, in clinical settings, culinary competence is geared toward avoiding UPFs as a strategy for glycemic control, while in the general population it focuses on improving diet quality.

### 4.1 Study population profile

Analysis of the sociodemographic and clinical profiles revealed notable differences between participants with type 1 diabetes and the healthy control group. The diabetes cohort had a higher mean age, whilst the healthy group included more students and young adults. Furthermore, a higher prevalence of low income was observed in the diabetes group, suggesting possible socioeconomic inequalities that could influence dietary habits and the ability to adopt healthy behaviors. These findings are consistent with previous studies conducted in Spain, which describe a similar profile in people with type 1 diabetes: a predominance of women, a mean age in their forties, and a heterogeneous socioeconomic distribution, with a significant proportion of individuals in low-income strata (47). Furthermore, the relationship between sociodemographic factors and eating behaviors has been extensively documented, highlighting that financial and educational constraints can influence the adoption of healthy cooking practices and increase reliance on convenience/pre-prepared food, both in the general population and among people with chronic conditions (48). Similarly, it has been noted that social inequalities affect not only the quality of the diet, but also perceptions of self-efficacy and culinary competence, which reinforces the need for interventions tailored to the socio-economic context (49–51).

In terms of metabolic control, the mean glycated hemoglobin observed in the cohort with diabetes was close to the targets recommended by international guidelines, although there was considerable variability between individuals. This pattern is consistent with that reported in national studies, which indicate that, despite advances in technology and diabetes education, challenges remain in achieving optimal glycemic control across the entire population (52, 53). Factors such as prolonged duration of the disease, therapeutic burden, and differences in access to resources may explain this variability.

Regarding T1D management, the results show that most participants with T1D use intensive insulin regimens, mainly through multiple daily injections or continuous subcutaneous infusion, along with high adoption of continuous glucose monitoring (CGM) technologies. This pattern reflects an advanced approach consistent with international recommendations, which promote intensive strategies and the use of technologies to optimize glycemic control (54).

### 4.2 Comparison of culinary skills between patients with type 1 diabetes and healthy controls

The findings indicate that people with T1D have higher levels of culinary competence than the healthy population, especially in health-oriented skills such as reading nutrition labels, modifying recipes, and preparing vegetables, suggesting that the demands of glycemic self-management promote food literacy and the acquisition of practical skills. This trend is consistent with research indicating that diet education and experience in managing chronic diseases promote greater development of culinary and planning skills (24, 55). Furthermore, recent literature confirms that culinary competence and food literacy are protective factors against the consumption of UPFs, whose high intake is associated with an increased risk of obesity, cardiovascular disease, and diabetes, even independently of the overall quality of the diet (23, 25). In this context, T1D represents a critical scenario, as reducing UPF contributes to improving glycemic stability and decreasing postprandial variability, which are essential for preventing long-term complications.

### 4.3 The effect of culinary competition on unhealthy eating habits, taking sociodemographic variables into account

The data confirm that high levels of culinary competence are associated with better indicators of dietary quality, including lower consumption of UPFs, less use of unhealthy sauces and fats, and less reliance on cooking methods such as frying. However, subgroup analysis reveals different patterns: in the T1D cohort, cooking skills were a significant predictor for avoiding UPFs, while no relationship was observed with the use of sauces or heavy cooking techniques, suggesting that dietary restrictions and nutritional education inherent in disease management already limit these practices. In contrast, in the healthy population, cooking skills were associated with lower intake of sauces and unhealthy cooking methods but did not influence the frequency of consumption of pre-cooked foods, indicating that this group prioritizes qualitative improvement of the diet rather than total exclusion of processed products. These findings are consistent with recent studies confirming that cooking skills and food education are protective factors against the convenience/pre-prepared food consumption and contribute to improving the overall quality of the diet (24, 56). Furthermore, in people with diabetes, dietary education and the need to plan carbohydrate intake may explain the stronger association with reduced convenience/pre-prepared food consumption, given that these products tend to be high in sugars and fats, which make glycemic control difficult (55).

Multivariate analysis showed that culinary competence remained an independent predictor of healthier eating habits even after adjusting for age, sex, educational level, and income, reinforcing its role as a practical skill with a direct impact on dietary quality. This is consistent with recent studies that have shown that culinary skills and food literacy are significantly associated with lower intake of convenience/pre-prepared food, even after controlling for sociodemographic variables (24).

### 4.4 Sociodemographic and behavioral analysis and characterization of latent culinary competence profiles (LPA)

Latent Profile Analysis revealed two clearly differentiated subgroups based on culinary competence: a minority profile with moderate skills, characterized by deficits in strategic planning and food preservation, and a majority profile of “culinary experts,” with high levels of technical and cognitive skills, including the preparation of vegetables, grains, and proteins, as well as the modification of recipes to improve their nutritional quality. This segmentation confirms that culinary competence is not homogeneous but rather distributed in patterns that reflect differences in experience, motivation, and exposure to food education. These results are consistent with recent research that has used classification models to examine heterogeneity in culinary skills, showing that profiles with higher competence are associated with better diet quality and lower convenience/pre-prepared food consumption (25, 57). Furthermore, poor culinary skills are associated with a greater reliance on processed foods and a lower adherence to healthy eating habits (58, 59).

In the context of type 1 diabetes, these differences are particularly relevant, given that planning and home preparation are essential components of glycemic control and complication prevention, reinforcing the need for personalized interventions that strengthen strategic competencies in subgroups with limited skills (3).

The sociodemographic analysis showed that the “Culinary Expert” it is determined by a lower frequency of convenience/pre-prepared food consumption, regardless of socioeconomic variables. The only significant sociodemographic difference was age, suggesting that accumulated experience and life stage may influence the development of culinary skills, consistent with studies that have documented a positive relationship between age and the frequency of home-cooked meals (60). Furthermore, recent research also suggests that age and prior experience influence the development of culinary skills, while young adults are more exposed to food environments dominated by convenience/pre-prepared food (24). The absence of differences based on gender, educational level, household income, or diabetes status reinforces the idea that culinary skills, rather than theoretical knowledge, are a key behavioral determinant in reducing the consumption of convenience/pre-prepared food (58, 59). These findings are particularly relevant in the context of T1D, where reducing UPFs contributes to improving glycemic stability and reducing postprandial variability, critical factors in preventing complications (54).

### 4.5 Limitations and strengths

The cross-sectional design employed is appropriate for the study’s objectives, which focus on characterizing associations between culinary competence and various eating behaviors in both individuals with type 1 diabetes and those without this condition. This approach is widely used in nutritional and behavioral research, especially when the aim is to explore relational patterns in real-world contexts and generate hypotheses for subsequent studies. However, the cross-sectional nature of the data precludes establishing causal or directional relationships, so the observed associations should be interpreted with caution. In particular, it cannot be ruled out that certain healthier eating behaviors may promote the development of greater culinary skills, or that both phenomena may evolve in parallel as part of broader processes of self-care and health literacy, especially in the context of a chronic disease such as type 1 diabetes. The sampling strategy, based on a combination of convenience and chain sampling, allowed for an adequate sample size and access to a specific population that is often underrepresented in population-based studies. However, this approach may have introduced self-selection bias, favoring the participation of individuals with a greater interest in nutrition, greater involvement in their self-care, and higher levels of health literacy. Furthermore, the overrepresentation of women and certain age groups limits the direct extrapolation of the results to the general population. Although the analyses were adjusted for relevant sociodemographic variables, these adjustments do not completely eliminate the limitations inherent in non-probability samples; therefore, the findings should be interpreted primarily in terms of internal validity and not as estimates of population prevalence.

On the other hand, the use of self-reported data is an appropriate and common methodological strategy in studies of this type, especially for assessing skills, cooking practices, and food consumption frequency. However, it is necessary to acknowledge the possibility of information bias, particularly social desirability bias, which could have led to an underestimation of convenience/pre-prepared food consumption or an overestimation of cooking skills and practices considered socially healthy. In this regard, an additional relevant limitation concerns the assessment of convenience/pre-prepared food consumption. Although the NOVA classification framework was adopted, intake was operationalized through a single question about the weekly frequency of consumption of “prepared foods.” This should be interpreted as an indirect, frequency-based indicator rather than a comprehensive assessment of UPFs intake as defined by the full NOVA system. This approach fails to capture in detail the degree of food processing at the ingredient or nutrient level, nor the inherent multidimensionality of this classification. Therefore, the results regarding UPFs consumption should be interpreted with caution.

Finally, although the study controlled for several key sociodemographic variables, the absence of other potentially relevant factors—such as available cooking time, work or family burden, and the characteristics of the food environment—limits the contextualization of the observed associations. This limitation is reinforced by the demographic composition of the cohort, as the healthy control group was younger and had a higher percentage of students compared to the group with type 1 diabetes. These differences in age and employment status can act as confounding variables in life-stage-sensitive eating behaviors. Furthermore, the sex distribution within the sample should be considered when assessing the generalizability of the results. Finally, selection bias cannot be ruled out, since individuals more interested in healthy eating and self-care may have been more motivated to participate, which could lead to an overestimation of average cooking competence. Overall, the inclusion of additional contextual variables in future studies would allow for a more nuanced understanding of the structural determinants underlying the relationship between cooking competence and diet quality.

Despite these limitations, the study has several important strengths. First, the large sample size and the inclusion of a well-characterized clinical group of people with type 1 diabetes alongside a comparison group stand out, allowing for the exploration of specific differences and patterns according to clinical status. Furthermore, a structured and previously validated instrument was used to assess culinary competence, complemented by variables that capture specific cooking practices and everyday eating behaviors. The use of multivariate models adjusted for key sociodemographic factors reinforces the robustness of the observed associations, and the application of Latent Profile Analysis provides an innovative approach to identifying relevant behavioral subgroups. Taken together, these strengths contribute to the study’s internal validity and strengthen its contribution to the growing body of evidence on the role of culinary competence as a behavioral determinant of diet quality, especially in the context of self-care in type 1 diabetes.

### 4.6 Areas for further research

The findings of this study open multiple opportunities to further explore the relationship between culinary competence, convenience/pre-prepared food consumption, and type 1 diabetes (T1D) management. First, longitudinal studies are needed to establish causal relationships between the development of culinary skills and sustained reduction in convenience/pre-prepared food consumption, as well as their impact on glycemic control and postprandial variability. Second, it would be relevant to evaluate the effectiveness of interventions based on practical culinary education, compared to strategies focused solely on the transmission of theoretical knowledge, in both clinical and community settings. Likewise, future research should explore the mediating role of food literacy and strategic meal planning in dietary adherence and the prevention of metabolic complications in people with T1D.

## 5 Conclusion

This study identifies a significant association between culinary competence and diet quality, as well as with a lower frequency of convenience/pre-prepared food consumption, a relationship that remained after adjusting for relevant sociodemographic variables. In people with type 1 diabetes, greater culinary competence was especially associated with a lower intake of convenience/pre-prepared food, while in the healthy population, this competence was mainly related to qualitative improvements in food preparation.

The findings support the incorporation of practical culinary education programs into both public health strategies and the clinical management of T1D, given their potential to reduce exposure to UPFs, improve glycemic control, and promote healthier eating patterns.

## Data Availability

The raw data supporting the conclusions of this article will be made available by the authors, without undue reservation.
